# Observations on the Structure and Diseases of the Testis; with Numerous Plates

**Published:** 1845-02

**Authors:** 


*Observations on the Structure and Disease of the Testis; with numerous plates.* By SIR ASTLEY COOPER, Bart., F. R. S., Sergeant Surgeon to the Queen, Consulting Surgeon to Guy's Hospital. From the second London Edition. Edited by BRANSBY B. COOPER, F. R. S., Surgeon to Guy's Hospital, Lecturer on Anatomy.

